# Supplementary material for: Ladder Use Ability, Behavior and Exposure by Age and Gender
Source: Geriatrics (Basel). 2024 May 10;9(3):61. doi: 10.3390/geriatrics9030061 (PMC11130934; doi:10.3390/geriatrics9030061)
Supplement: Supplementary file 1 [file geriatrics-09-00061-s001.zip › geriatrics-2958357-supplementary.pdf]

Ladder Use History Survey

Erika M Pliner[1,2,], Daina L Sturnieks[1,3], Stephen R Lord[1,4]

[1]Falls, Balance and Injury Research Centre, Neuroscience Research Australia, Sydney, NSW, AUS
[2]Department of Bioengineering, University of Pittsburgh, Pittsburgh, PA, USA
[3]School of Medical Sciences, University of New South Wales, Sydney, NSW, AUS
[4]School of Public Health and Community Medicine, University of New South Wales, Sydney, NSW, AUS

Bold text denotes text in the survey. Italicized text denotes survey notes.

This survey will ask you questions about your ladder use. Some ladder use questions will be specific to a ladder type. Please see the descriptions and figures below for ladder types.

Step ladders are ladders with flat rectangular steps. A lot of step ladders fold together for storage. A step ladder can be either an A-frame ladder or step stool ladder. You may use these ladders inside to reach a high shelf or outside during house work.

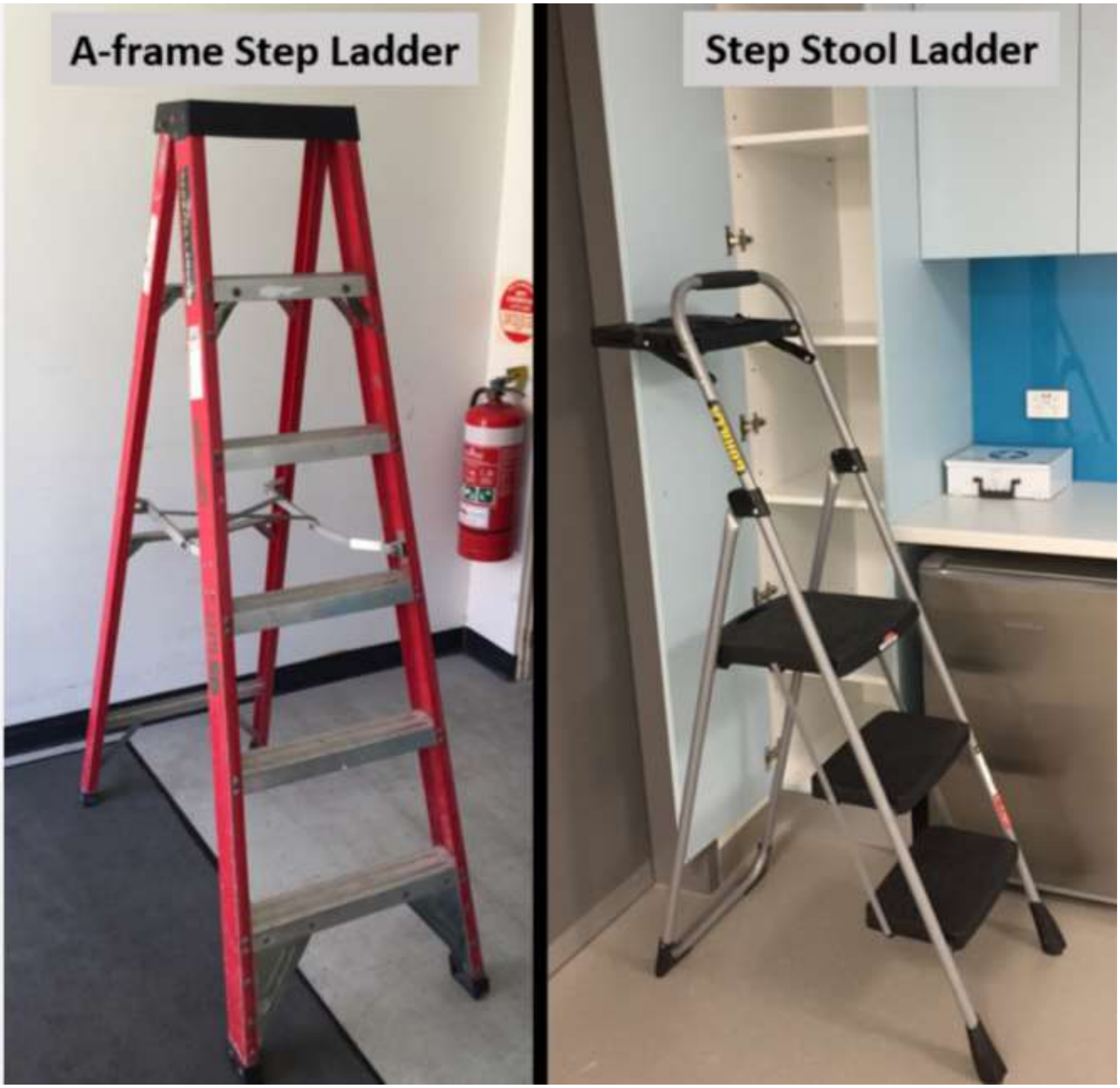

Inclined (also referred to as, straight or slanted) ladders are ladders you lean against a wall or a stable surface. Inclined ladders can be made of single or multiple sections (multi-section ladders are commonly referred to as, extension ladders). These ladders are often used outside to reach higher levels.

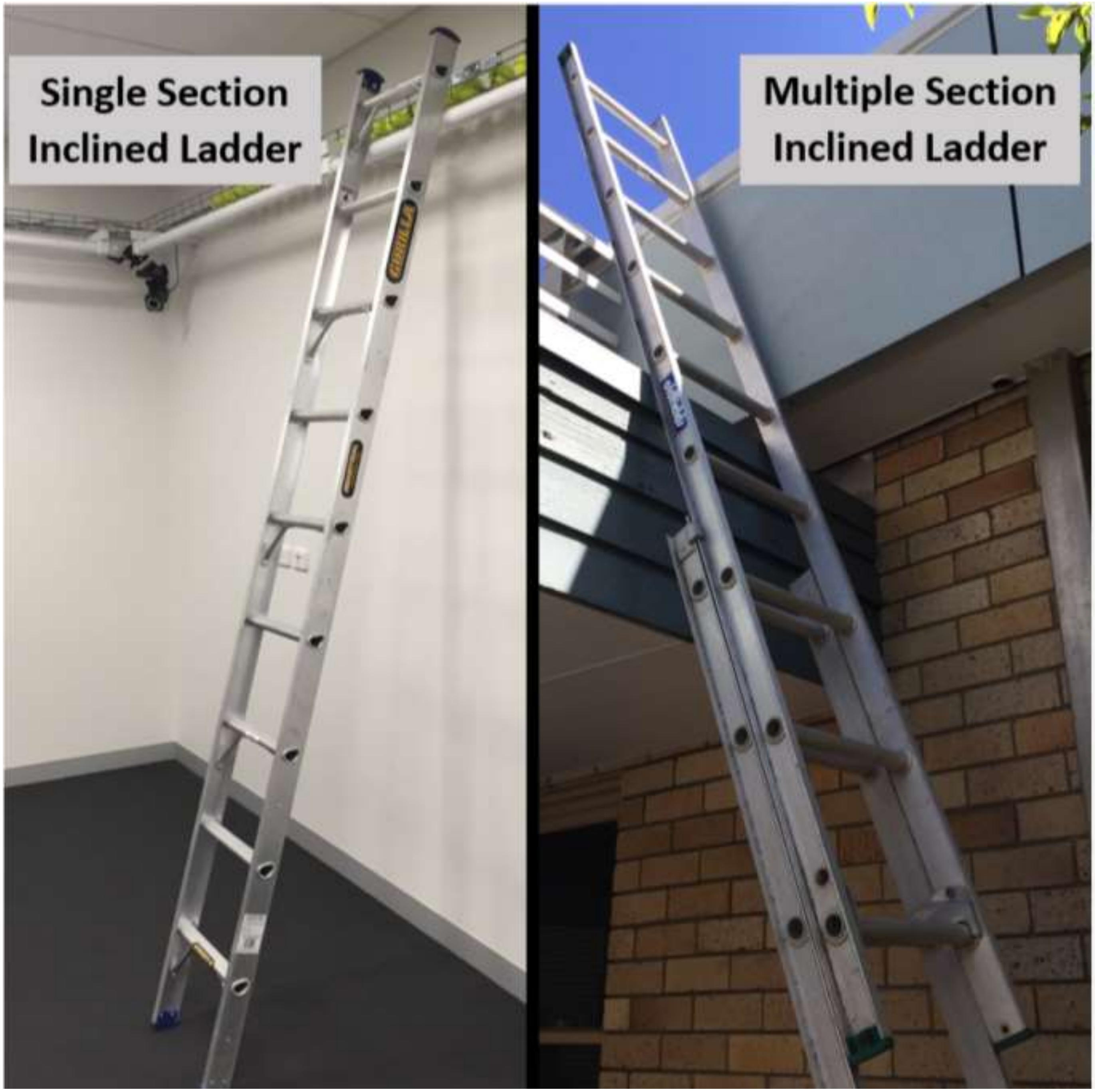

A fixed ladder is any type of ladder that is attached to something else. You may commonly use these types of ladders to climb to the attic, get into or out of a campervan/truck/boat, or get into or out of the water.

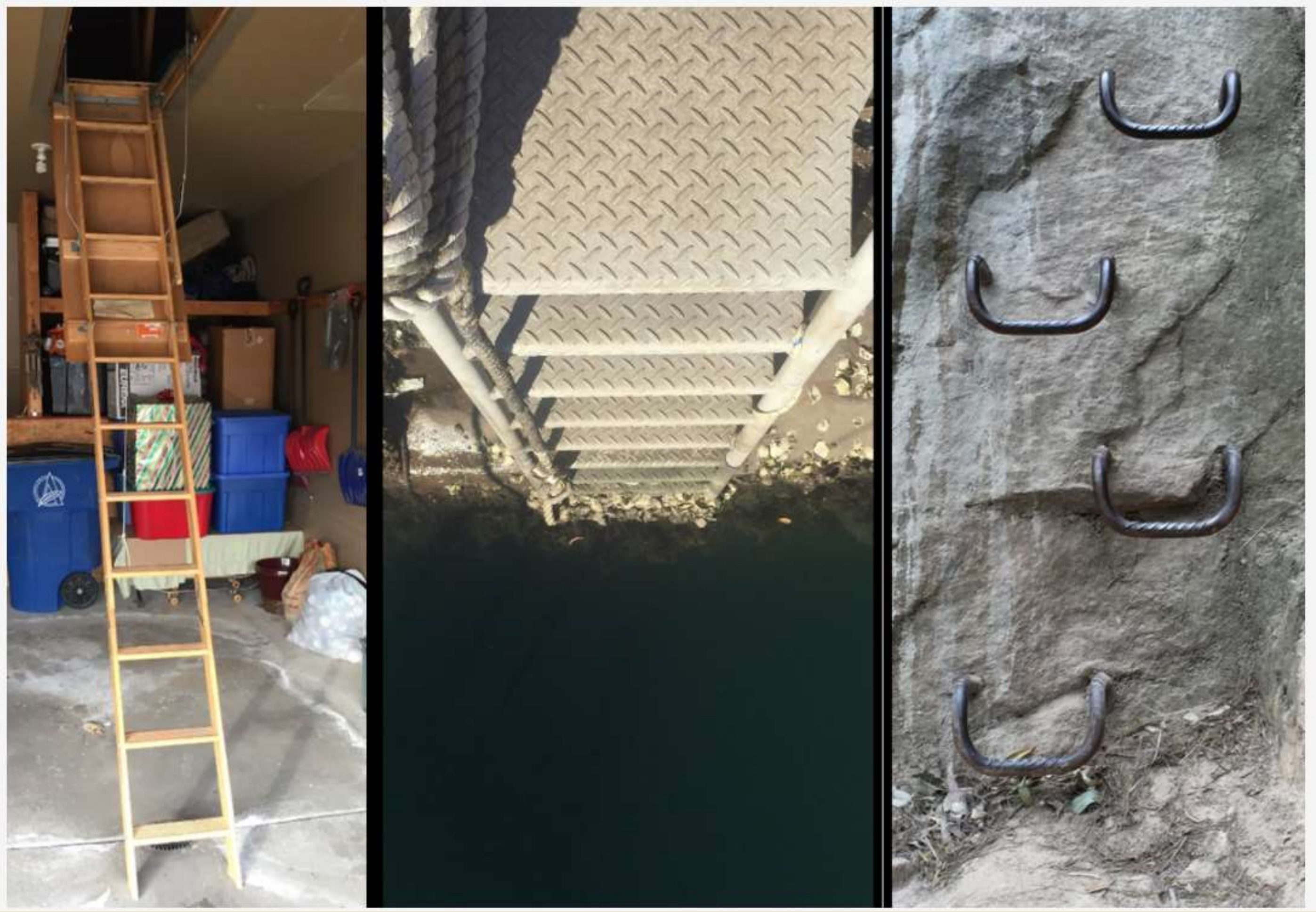

Ask the following question to everyone.

I use this type of ladder	daily	few times a week	weekly	monthly	few times a year	once a year	never
**Step Ladder**	○	○	○	○	○	○	○ reset
**Inclined (Slanted) Ladder**	○	○	○	○	○	○	○ reset
**Fixed Ladder**	○	○	○	○	○	○	○ reset

If any ladder was selected, ask the following question.

How concerned are you of falling from a ladder?

- ○ Not concerned
- ○ Slightly concerned
- ○ Moderately concerned
- ○ Very concerned

reset

If the no ladders are ever used, ask the following question.

Please describe or list any reasons why you do not climb ladders.

Expand

Ask the following questions for the corresponding ladders that are used.

**The highest step I climb to on this ladder is**	1st step	below halfway	halfway	above halfway
**Step Ladder**	○	○	○	○ reset
**Inclined (Slanted) Ladder**	○	○	○	○ reset
**Fixed Ladder**	○	○	○	○ reset

**I use this ladder for the following (check all that apply)**	Changing a light bulb	Cleaning the gutters	Washing the windows	Cutting branches or picking fruit	Getting objects from the attic	Decorating	Other
**Step Ladder**	☐	☐	☐	☐	☐	☐	☐
**Inclined (Slanted) Ladder**	☐	☐	☐	☐	☐	☐	☐
**Fixed Ladder**	☐	☐	☐	☐	☐	☐	☐

**I use this ladder (check all that apply)**	Inside	Outside	At home	Places other than my home
**Step Ladder**	☐	☐	☐	☐
**Inclined (Slanted) Ladder**	☐	☐	☐	☐
**Fixed Ladder**	☐	☐	☐	☐

Ask the following questions to everyone.

Compared to others, I believe my ladder use and climbing abilities are

	below average	slightly below average	average	slightly above average	above average
**Step Ladder**	○	○	○	○	○ reset
**Inclined (Slanted) Ladder**	○	○	○	○	○ reset
**Fixed Ladder**	○	○	○	○	○ reset

Compared to others MY AGE, I believe my ladder use and climbing abilities are

	below average	slightly below average	average	slightly above average	above average
**Step Ladder**	○	○	○	○	○ reset
**Inclined (Slanted) Ladder**	○	○	○	○	○ reset
**Fixed Ladder**	○	○	○	○	○ reset

Ask the following questions to individuals who use step ladders.

Please check all boxes that describe your STEP LADDER use

Please refer to this diagram for the question below

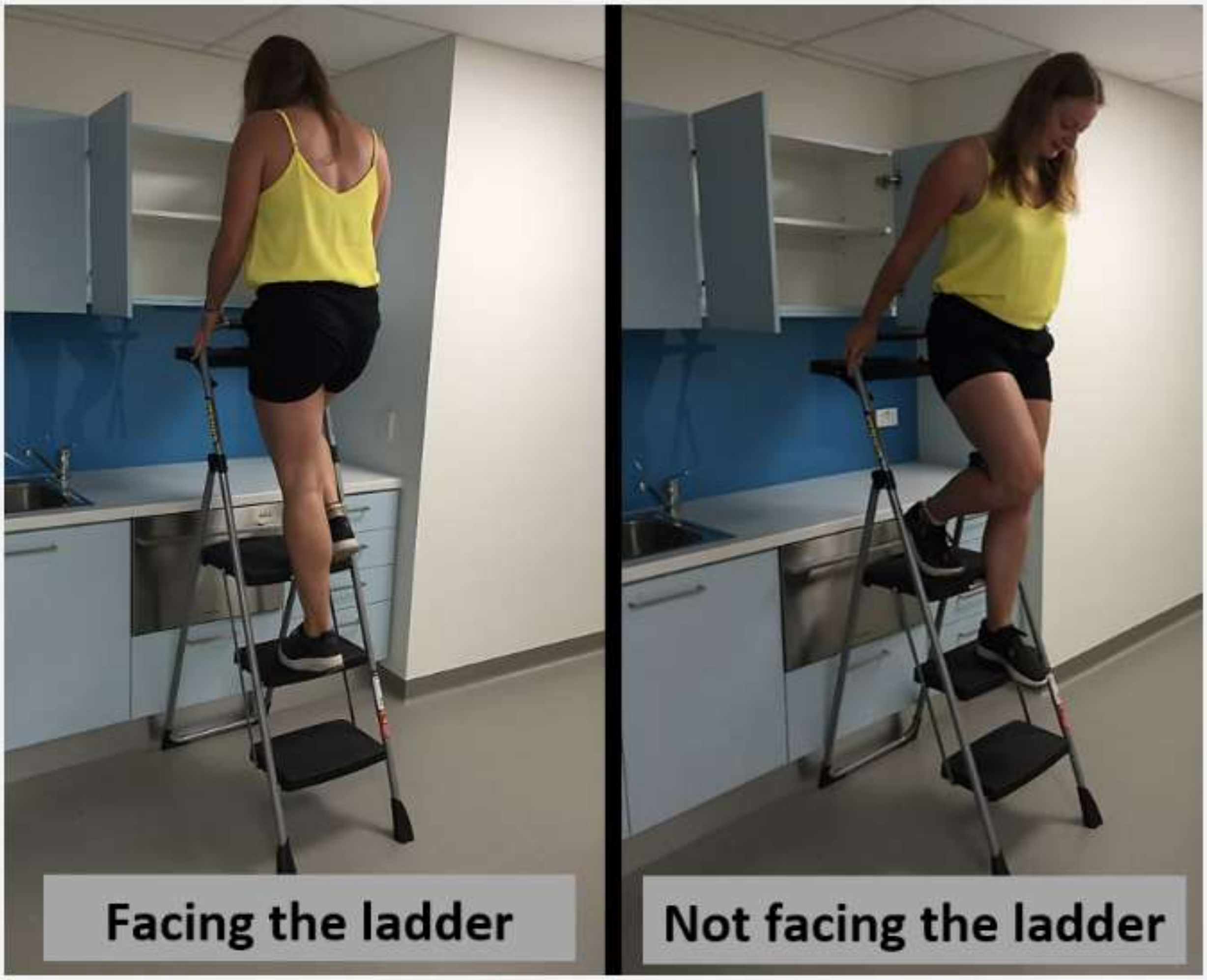

**It's common for me to descend a step ladder (check all that apply)**	☐ facing the ladder<br>☐ not facing the ladder
**It's common for me to climb a step ladder (check all that apply)**	☐ without using my hands<br>☐ with only one hand on the ladder<br>☐ with both hands on the ladder<br>☐ moving my feet one step/rung at a time<br>☐ moving my feet more than one rung/step at a time
**It's common for me to climb a step ladder (check all that apply)**	☐ without items/tools in my hand(s)<br>☐ with items/tools in my hand(s)<br>☐ with a tool belt
**The type of step ladders I climb are (check all that apply)**	☐ A-frame ladders (step ladder with 5 or more steps)<br>☐ step stool ladders (step ladder with 3 or less steps)

Ask the following question to individuals who use A-frame ladders.

Please refer to this diagram for the A-frame step ladder question below

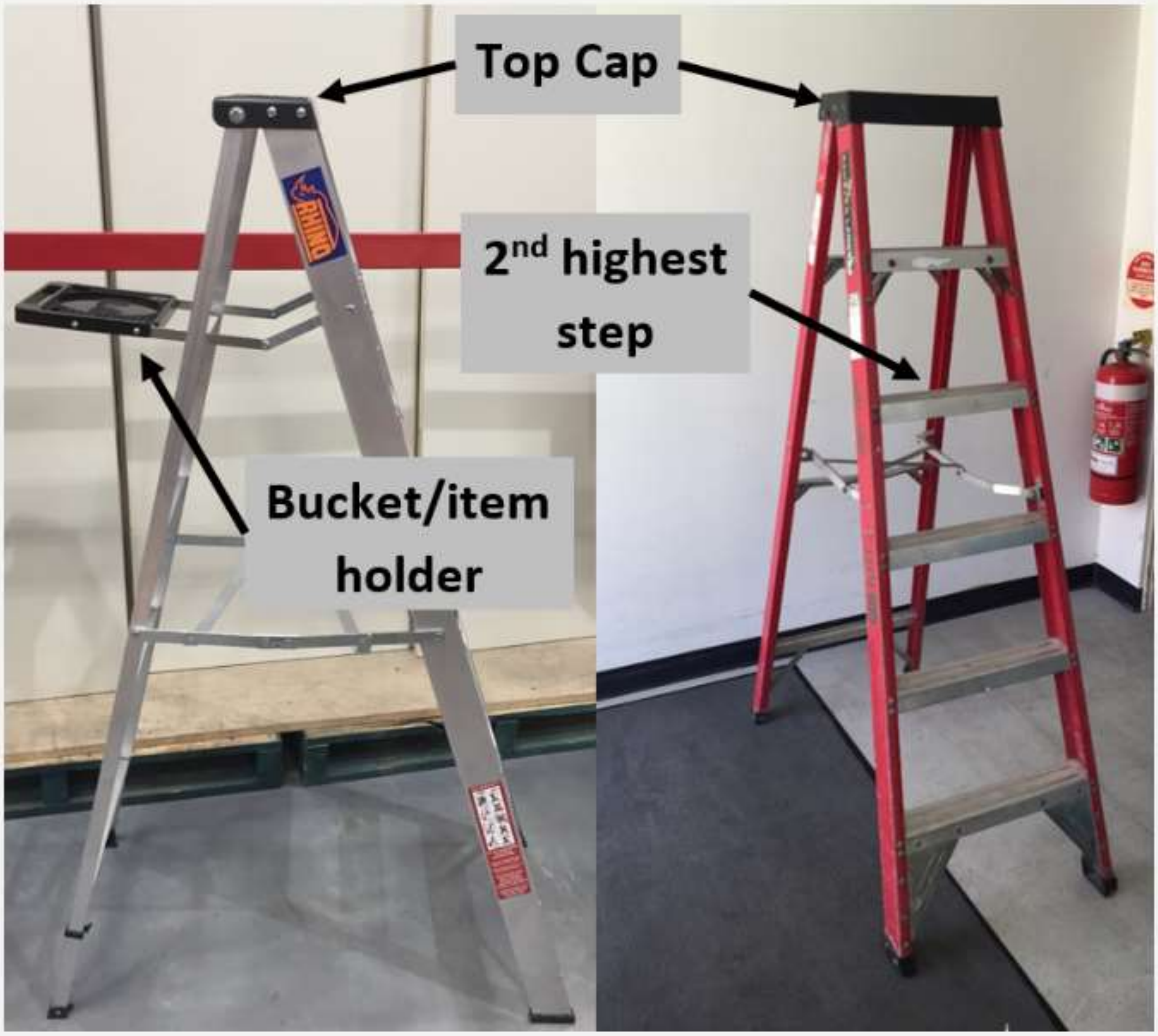

On A-frame ladders, I have (check all that apply)

- ☐ stood higher than the second highest step
- ☐ stood on the top cap of the ladder
- ☐ straddled the ladder (one foot on each side of the A-frame)
- ☐ stood or sat on the bucket/item holder

Ask the following question to individuals who use step stool ladders.

Please refer to this diagram for the step stool ladder question below

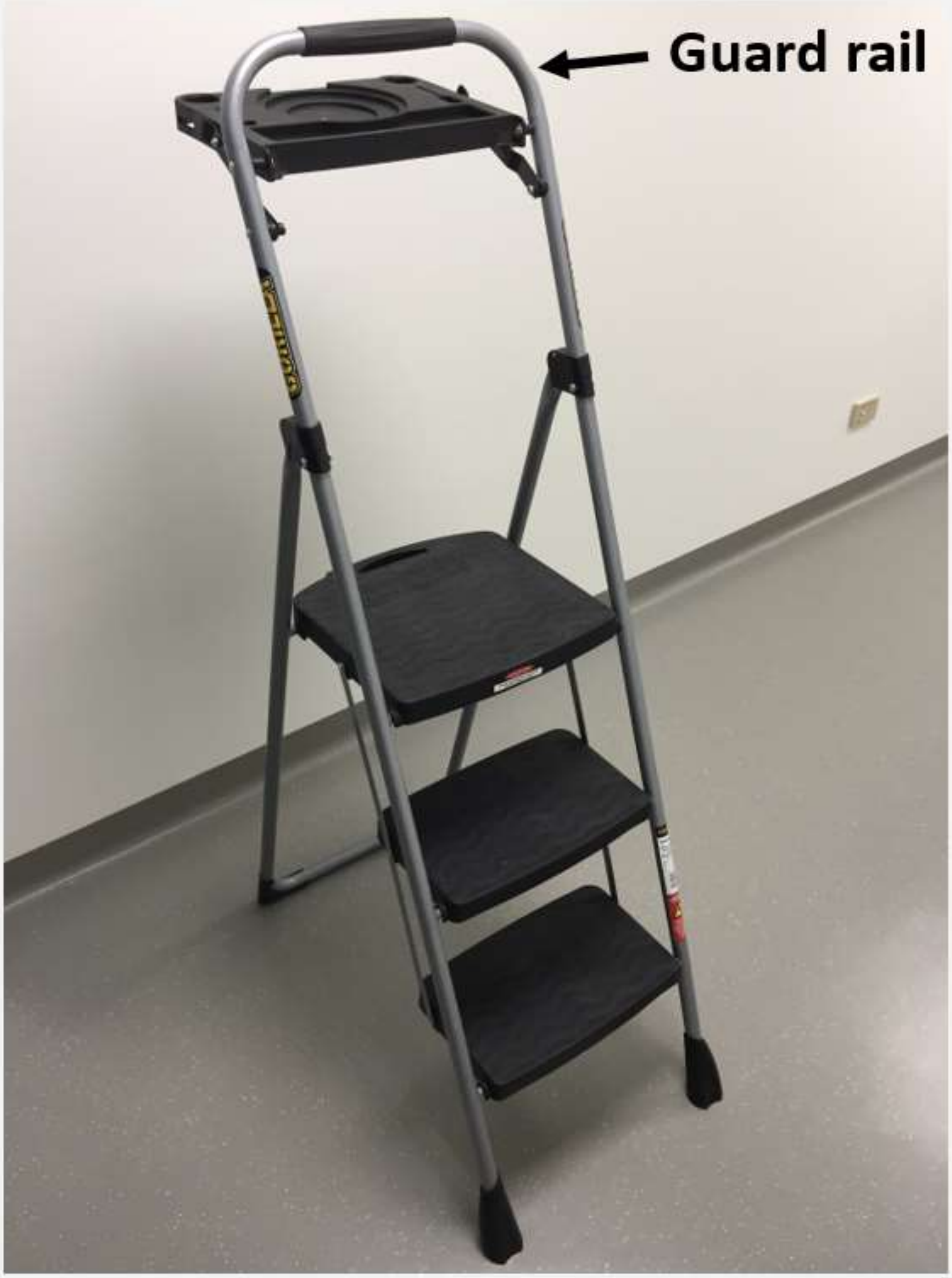

While I was on a step stool ladder,
(check all that apply)

- ☐ the guard rail has been above my knees
- ☐ the guard rail has been below my knees
- ☐ the guard rail was not been present
- ☐ I held onto the guard rail
- ☐ I did not hold onto the guard rail (if present)

Ask the following questions to individuals who use inclined ladders.

Please check all boxes that describe your INCLINED (SLANTED) LADDER use

Please refer to this diagram for the question below

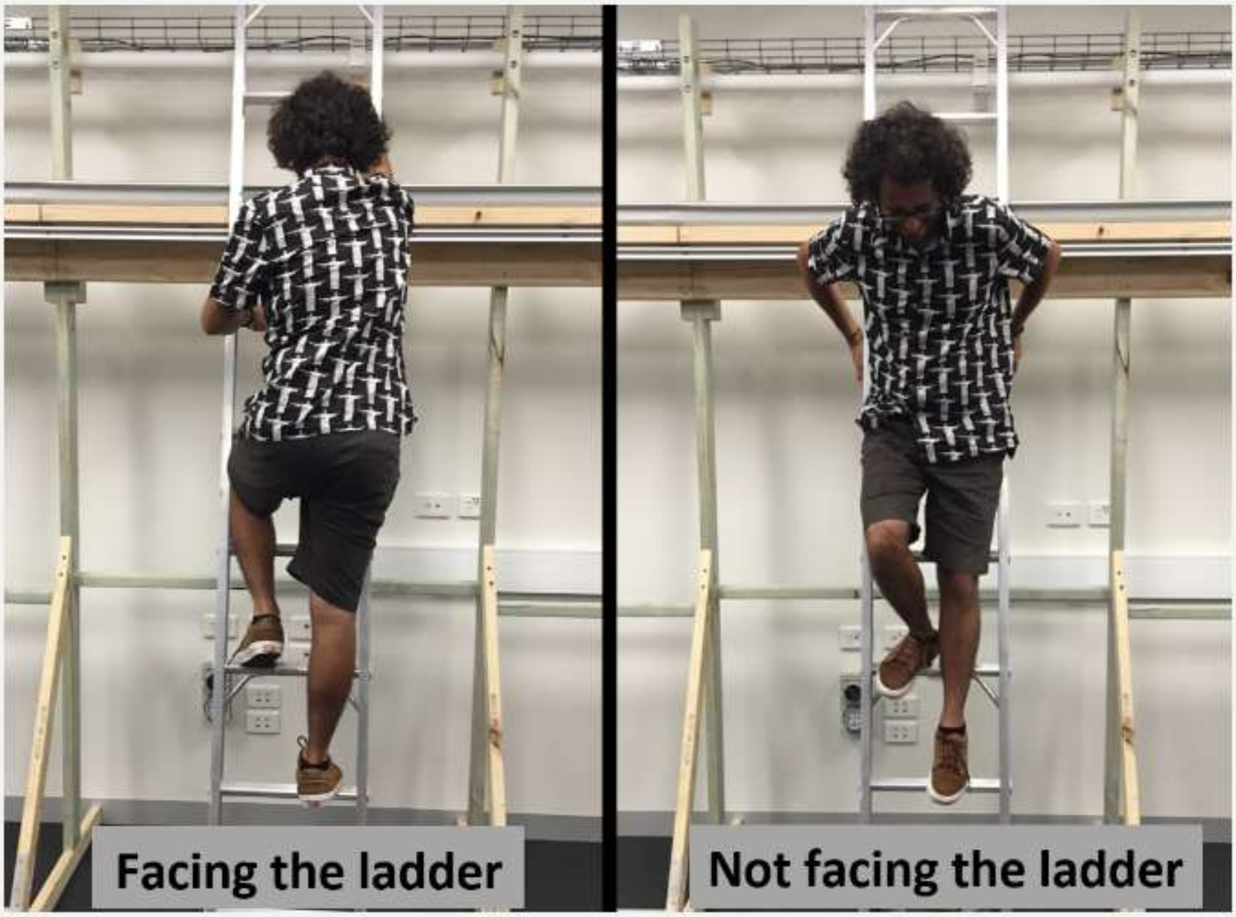

It's common for me to descend inclined (slanted) ladders (check all that apply)

- ☐ facing the ladder
- ☐ not facing the ladder

It's common for me to climb an inclined (slanted) ladder (check all that apply)

- ☐ without using my hands
- ☐ with only one hand on the ladder
- ☐ with both hands on the ladder
- ☐ moving my feet one step/rung at a time
- ☐ moving my feet more than one rung/step at a time

It's common for me to climb an inclined (slanted) ladder (check all that apply)

- ☐ without items/tools in my hand(s)
- ☐ with items/tools in my hand(s)
- ☐ with a tool belt

On inclined (slanted) ladders, I have stood (check all that apply)

- ☐ below the third highest rung/step
- ☐ on or above the third highest rung/step
- ☐ on the top rung/step

Ask the following questions to individuals who use any type of ladder.

Please check all boxes that describe your general (step, inclined or fixed) ladder use

Please refer to the diagrams for the question below

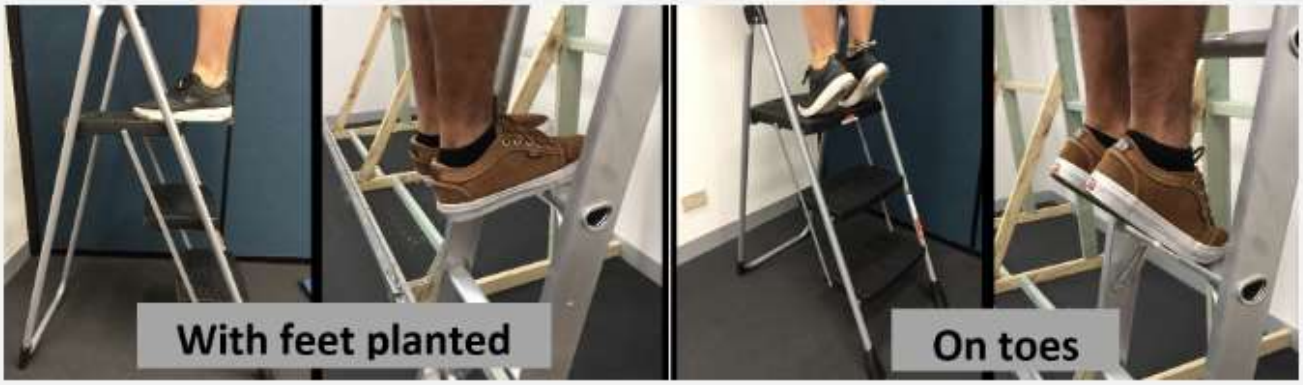

It's common for me to stand on a ladder (check all that apply)

- ☐ with my feet planted
- ☐ on my toes

Please refer to the diagrams for the question below

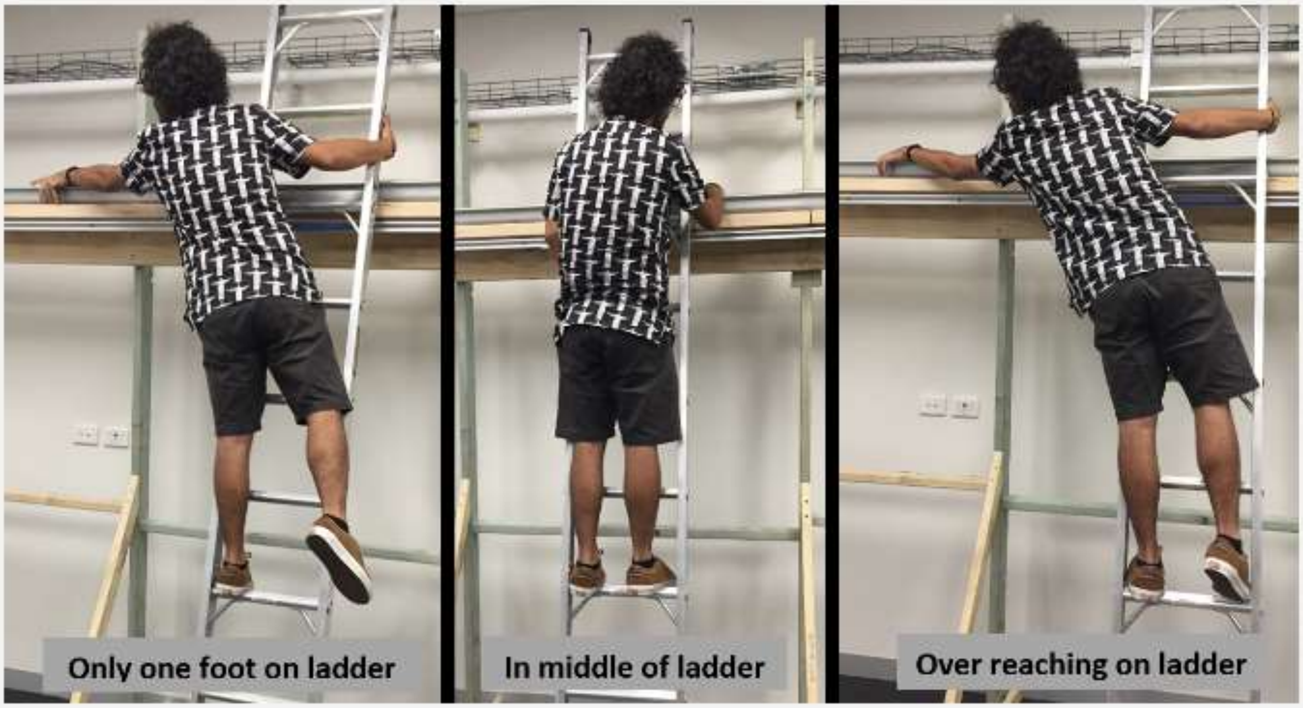

It's common for me to stand on a ladder (check all that apply)

- ☐ with only one foot on the ladder
- ☐ while in the middle of the ladder
- ☐ while over reaching*

*over reaching is reaching to your absolute limit

Please refer to the diagrams for the question below

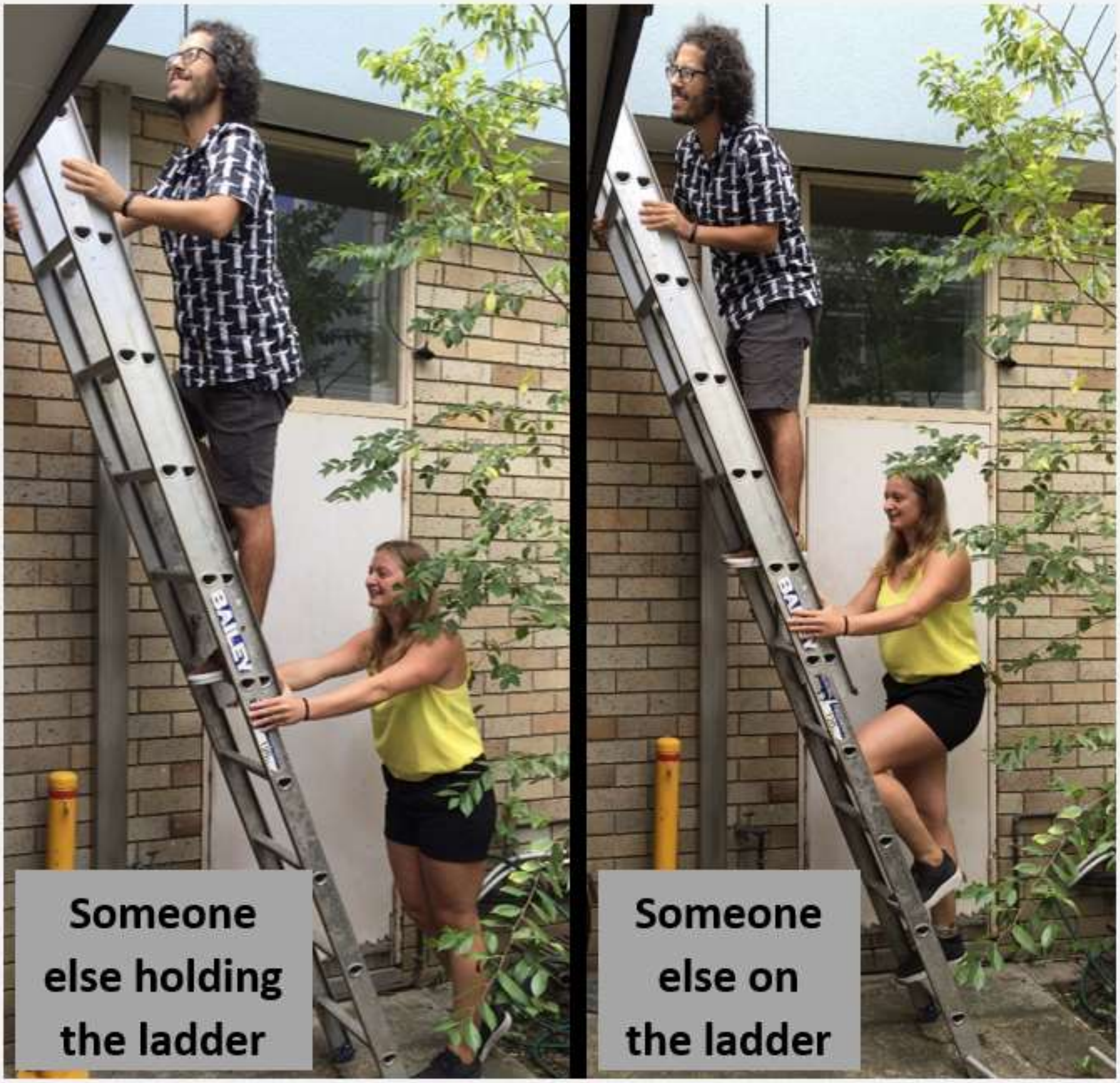

It's common for me to stand on a ladder
(check all that apply)

- ☐ without someone holding the ladder
- ☐ with someone else holding the ladder
- ☐ with someone else on the ladder

Please refer to this diagram for the question below

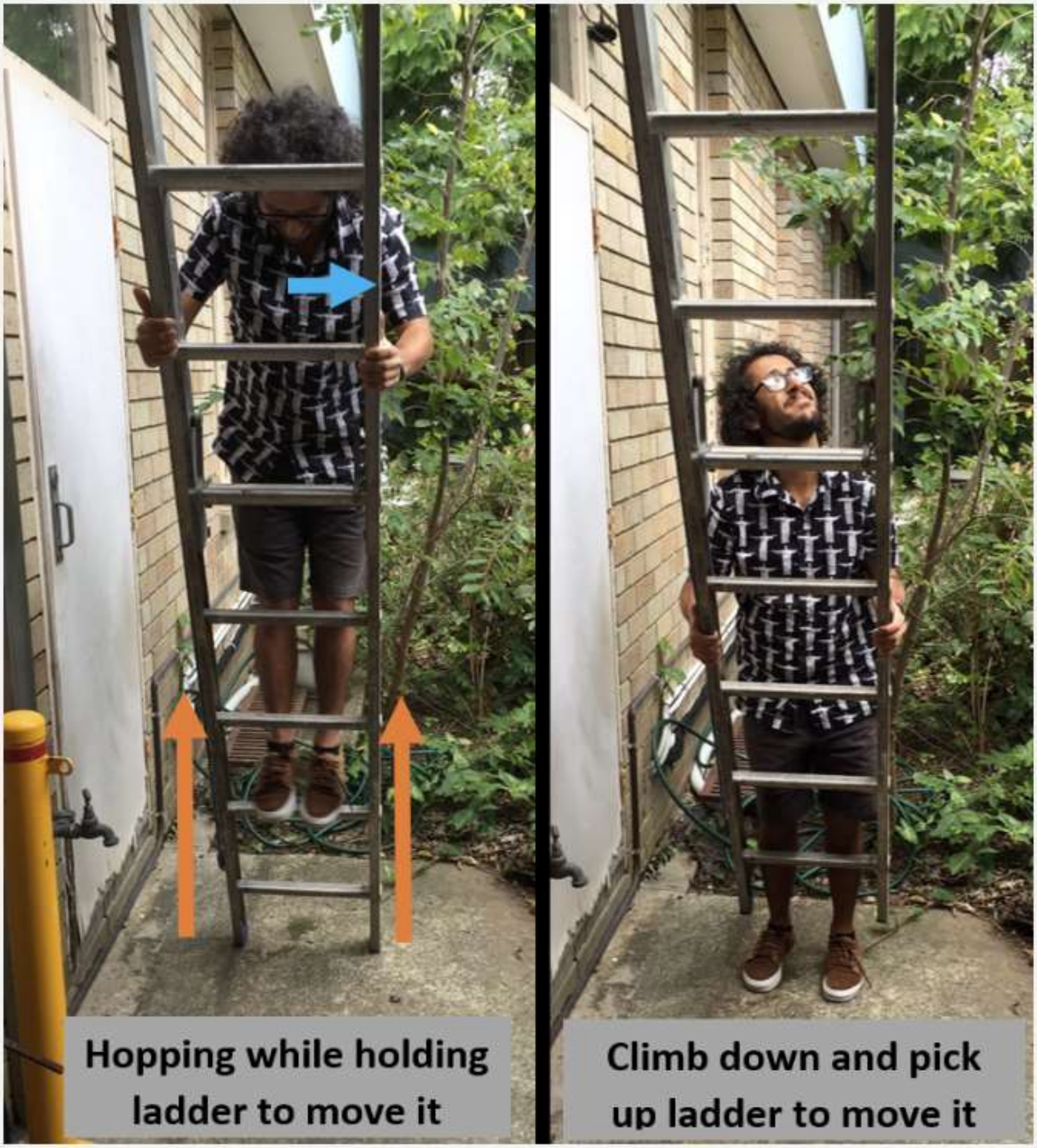

**I have moved a ladder by (check all that apply)**	☐ hopping while holding the ladder to move it<br>☐ climbing down the ladder and picking it up to move it
**I have climbed a ladder when I was (check all that apply)**	☐ fatigued<br>☐ tired<br>☐ dizzy, light headed or faint<br>☐ sick

Ask the following questions to everyone.

Ladder fall history	
**In the last 5 years, have you fallen from a ladder?**	◯ Yes<br>◯ No<br>reset

Ask the following questions for individuals who respond 'Yes' to falling from a ladder in the last 5 years.

**What type of ladder did you fall off?**	◯ Step Ladder<br>◯ Inclined (Slanted) Ladder<br>◯ Fixed Ladder<br>reset
**Was the fall**	◯ with the ladder<br>◯ from the ladder<br>◯ not sure<br>reset<br>A fall with the ladder is when the ladder falls with you. A fall from the ladder is when you fall from the ladder, but the ladder remains standing.
**What was your movement on the ladder at the time of the fall?**	◯ standing/working<br>◯ reaching<br>◯ ascending<br>◯ descending<br>◯ transitioning onto/from ladder<br>◯ mechanical failure of the ladder<br>◯ not sure<br>reset
**What caused the fall?**	◯ slipped on rung/step<br>◯ misstepping a rung/step<br>◯ lost balance<br>◯ ladder tipped<br>◯ ladder base slipped<br>◯ not sure<br>reset
**The fall was due to**	◯ improper ladder setup<br>◯ unsafe ladder use/climbing<br>◯ interference from an external object<br>◯ not sure<br>reset
**Reason for ladder use or activity being performed on the ladder**	
**Has the fall affected your ladder climbing abilities?**	◯ Yes<br>◯ No<br>reset
**Has the fall changed your behaviour on a ladder?**	◯ Yes<br>◯ No<br>reset
